# Vitamin B12 Deficiency-Induced Massive Schistocytosis and Hemolytic Anemia Due to Pseudo-Thrombotic Microangiopathy: A Case Report

**DOI:** 10.7759/cureus.80855

**Published:** 2025-03-19

**Authors:** Masahiro Manabe, Naoyuki Inano, Yuuji Hagiwara, Ki-Ryang Koh

**Affiliations:** 1 Hematology, Osaka General Hospital of West Japan Railway Company, Osaka, JPN; 2 Clinical Laboratory, Osaka General Hospital of West Japan Railway Company, Osaka, JPN

**Keywords:** histogram, megaloblastemia, pseudo-thrombotic microangiopathy, schistocyte, vitamin b12 deficiency

## Abstract

A 37-year-old male presented with complaints of fatigue, weight loss, and decreased appetite. Laboratory examinations revealed hemolytic anemia, an elevated lactate dehydrogenase (LDH) level, and marked schistocytosis. His other laboratory hematological findings included nucleated red blood cells with abnormal morphologies in the peripheral blood, as well as a small peak that was indicative of schistocytes on a red blood cell histogram. A bone marrow examination demonstrated megaloblastic changes; therefore, a preliminary diagnosis of vitamin B12 deficiency was made. Intramuscular vitamin B12 injections were started, and it was later reported that the patient’s serum vitamin B12 level was below the reference range. Hence, the diagnosis of vitamin B12 deficiency was confirmed. Thereafter, the hemolysis and anemia improved within five weeks. It has been reported that megaloblastic anemia accompanied by schistocytosis demonstrates similar laboratory hematological findings to thrombotic microangiopathy (TMA) and is therefore called pseudo-TMA. In such cases, it is important to identify and rule out other concomitant abnormalities based on laboratory findings, to make a prompt and accurate diagnosis and choose an appropriate treatment strategy.

## Introduction

Microangiopathic hemolytic anemia (MAHA) is a condition in which erythrocytes are mechanically restricted when passing through small blood vessels, resulting in erythrocyte degradation and hemolysis. MAHA may occur in isolation due to direct effects on red blood cells, such as trauma caused by mechanical heart valves, infections (e.g., malaria), or march hemoglobinuria, but it more commonly occurs as a component of thrombotic microangiopathy (TMA). TMA has various etiologies, which share similar laboratory and clinical characteristics. Determining the underlying etiology of TMA is critical for ensuring that appropriate treatment can be administered. Thrombotic thrombocytopenic purpura (TTP) is a life-threatening condition arising from primary hemostatic abnormalities. Although TTP is the first diagnosis suspected when a patient presents with hemolytic anemia and thrombocytopenia accompanied by schistocytosis, other diseases should be considered as well. In vitamin B12 deficiency-induced anemia, schistocytes are rarely observed and laboratory findings similar to those of TMA are seen; therefore, it is called pseudo-TMA [1]. 

Pseudo-TMA is characterized by hemolytic anemia, thrombocytopenia, schistocytosis, elevated lactate dehydrogenase (LDH) levels, and low reticulocyte production [2,3]. Regarding treatment, it is important to consider vitamin B12 deficiency as a cause because the treatments for and prognosis of vitamin B12 deficiency-induced pseudo-TMA and TTP are very different. Due to the high morbidity rate associated with delays in the treatment of TTP, patients are emergently managed with plasma exchange and may require admission to the ICU. On the other hand, pseudo-TMA associated with vitamin B12 deficiency can be treated with vitamin supplementation. We report a case of pseudo-TMA caused by vitamin B12 deficiency.

## Case presentation

A 37-year-old male was referred to our hospital due to a month-long history of fatigue, appetite loss, and body weight loss. A physical examination revealed pallor and icterus. The surface lymph nodes, liver, and spleen were not palpable. He had no relevant medical history and was not taking any medication. No neurological abnormalities were seen. Although he was not a vegan or a strict vegetarian, he only ate meat or seafood about once a month. He was found to have hemolytic anemia (hemoglobin concentration: 6.2 g/dL; mean corpuscular volume (MCV): 117.4 fL; reticulocytes: 44.8×10^9^/L; platelets: 105×10^9^/L; white blood cells: 4.0×10^9^/L; LDH: 3115 U/L; total bilirubin: 2.3 mg/dL; direct bilirubin: 0.8 mg/dL; and haptoglobin: <10 mg/dL). Direct and indirect Coombs tests were negative.

**Figure 1:**
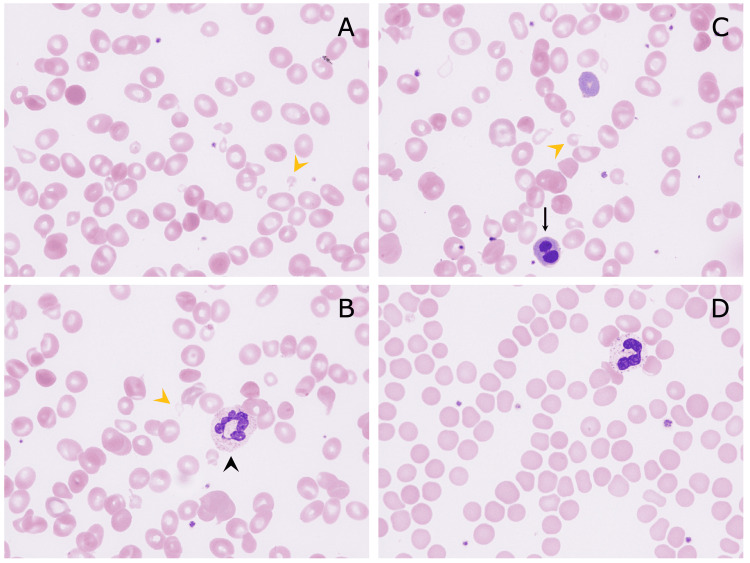
Peripheral blood smear (May-Giemsa staining, original magnification ×1000) Marked schistocytosis was seen at the initial presentation (A-C; yellow arrowheads, schistocytes). Hyperfragmented neutrophils (B, arrowhead) and nucleated red blood cells (C, arrow) were also observed. (D) No schistocytes were detected at five weeks after the initial diagnosis

**Figure 2:**
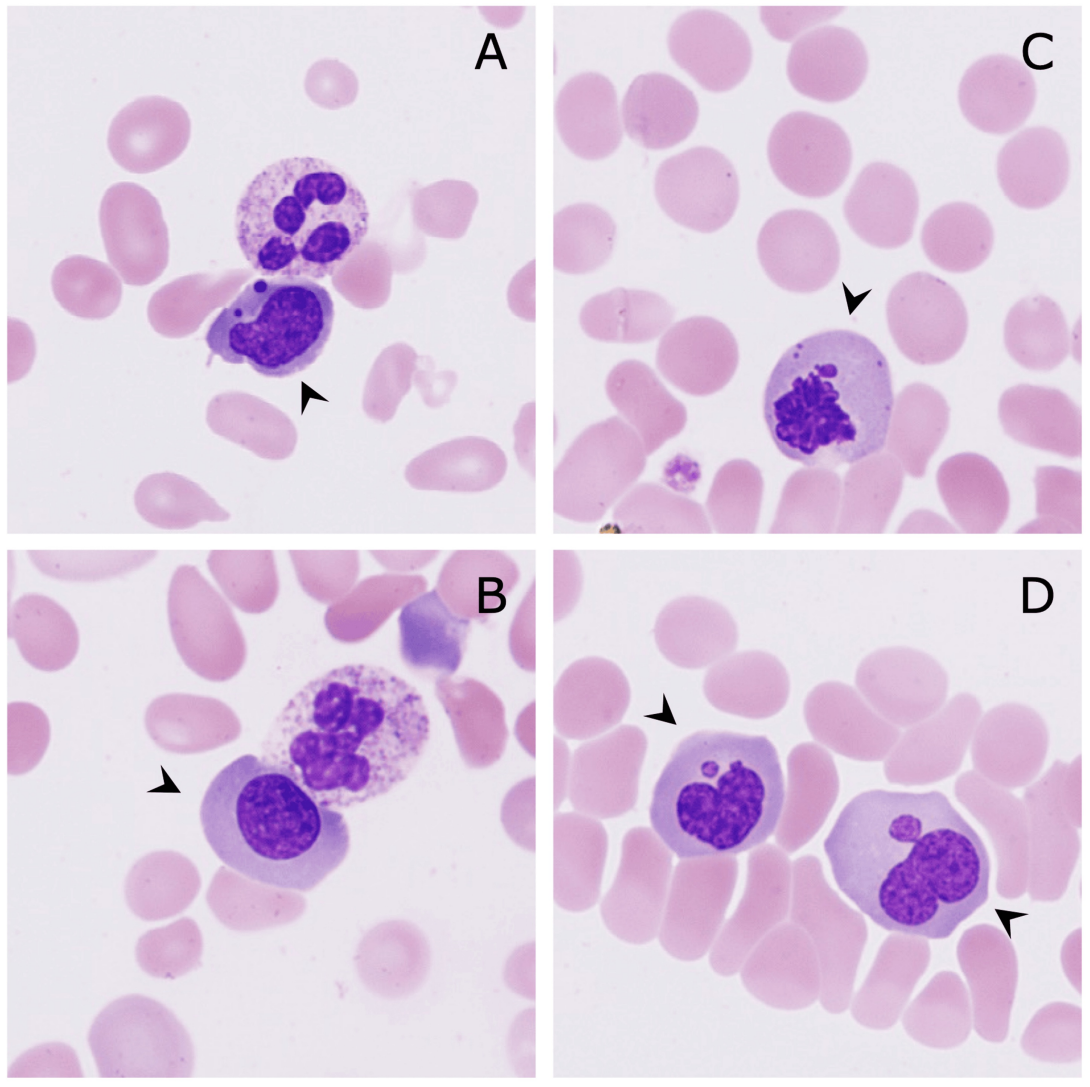
Nucleated red blood cells (May-Giemsa staining, original magnification ×1000) Dysplasia, including nuclear fragmentation (A), megaloblasts (B), and nuclear atypia (C, D), was seen

The patient's renal function markers were within the normal ranges (blood urea nitrogen: 10 mg/dL; serum creatinine: 0.72 mg/dL; estimated glomerular filtration rate: 98.6). A peripheral blood film revealed marked schistocytosis (Figures 1A-1C); a reticulocyte frequency of 3.07%; and nucleated red blood cells (6/100 white blood cells) with dysplasia, including megaloblastic changes that were suggestive of vitamin B12 deficiency (Figure 2). A Sysmex XN-2000 automated hematological analyzer (Sysmex, Kobe, Japan) was used to count nucleated red blood cells [4]. Furthermore, we manually calculated the nucleated red blood cell count to verify the accuracy of the automated count, and both counts were similar [5].

**Figure 3:**
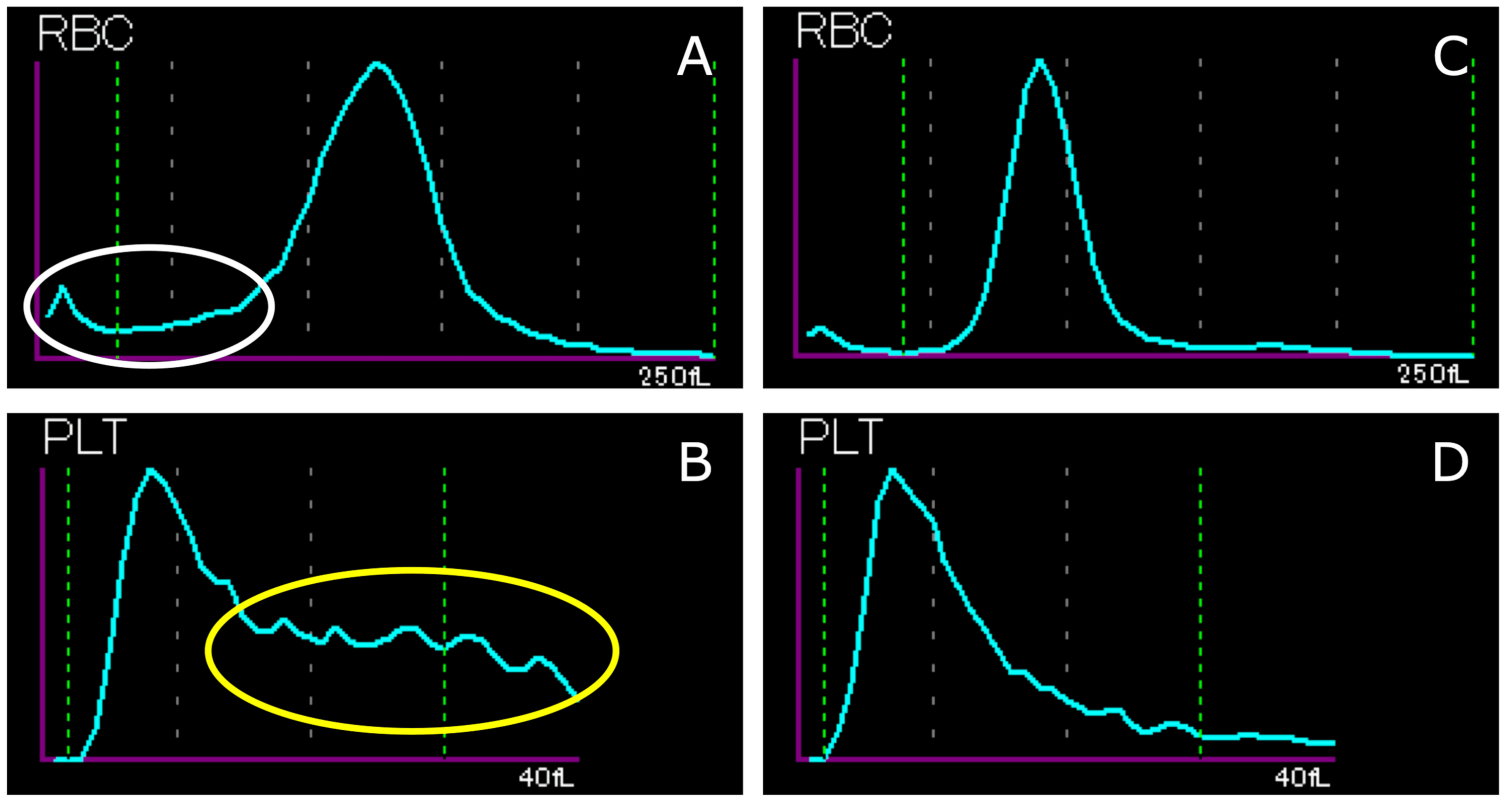
Red blood cell histogram (A) and platelet histogram (B) obtained at the initial diagnosis using an automated blood cell counter (XN-3000, Sysmex Corporation, Kobe, Japan) A small peak around 10 fL, which was linked to the main population and was indicative of smaller red blood cells (A, white circle), and an abnormal distribution that was suggestive of schistocytes (B, yellow circle) were seen. Five weeks after the initial presentation, the red blood cell histogram (C) and platelet histogram (D) no longer had abnormal distributions

In addition, a red blood cell volume (in fL) histogram demonstrated a small peak around 10 fL, and a platelet histogram produced using the impedance method showed an abnormal distribution due to the presence of schistocytes (Figure 3).

No remarkable abnormalities were seen in coagulation tests, except for a slightly elevated D-dimer level (prothrombin time-international normalized ratio: 1.17; activated partial thromboplastin time: 29.5 sec; fibrinogen: 256 mg/dL; fibrin degradation products: 3.1 μg/mL; and D-dimer: 1.8 μg/mL). In addition, no iron deficiency was seen (iron: 255 μg/dL; total iron-binding capacity: 278 μg/dL; ferritin: 722.1 ng/mL).

**Figure 4:**
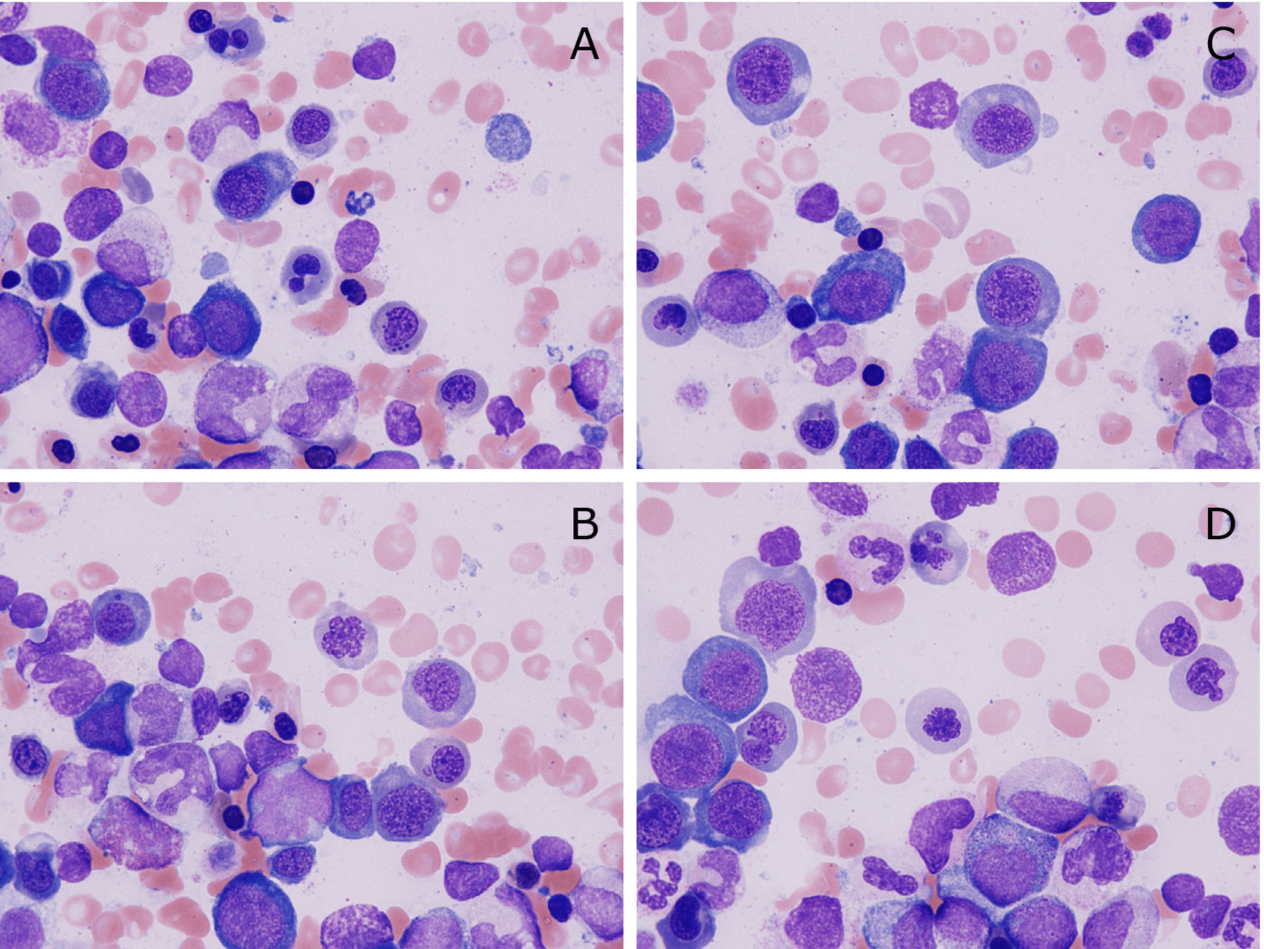
Bone marrow cells (May-Giemsa staining, original magnification ×1000) Marked megaloblastic changes throughout maturation stages were seen (A-D)

Although a diagnosis of TTP was considered due to the presence of hemolytic anemia and massive schistocytosis, a bone marrow examination, which was performed to definitively rule out cancer-related TMA [6], showed a hypercellular bone marrow with marked megaloblastic changes (fine chromatin structures were evenly distributed throughout the nucleus), which were similar to those seen on the peripheral blood smear and suggested vitamin B12 deficiency (Figure 4). In addition to the megaloblastic changes, further morphological abnormalities, including hypersegmented neutrophils and giant neutrophils in the myeloid series, irregular nuclei and multinucleated erythroblasts (dyserythropoiesis), and separated multinucleated megakaryocytes (dysmegakaryopoiesis), were seen; however, no chromosomal abnormalities suggesting myelodysplastic neoplasms were found.

The patient’s laboratory values on the second hospital day demonstrated thrombocytopenia and indicated that the anemia had worsened (platelets: 64×10^9^/L; hemoglobin concentration: 5.8 g/dL). A red blood cell transfusion was administered due to severe dyspnea on effort, and then we treated the patient with intramuscular cyanocobalamin, based on a suspicion of vitamin B12 deficiency. We considered measuring the patient’s A disintegrin and metalloproteinase with a thrombospondin type 1 motif, member 13 (ADAMTS13) activity levels to completely rule out TTP; however, we decided against it as we suspected vitamin B12 deficiency more strongly based on the results of the bone marrow examination. On the fifth hospital day, the patient’s serum level of vitamin B12 was reported as 76 pg/mL (reference range: 233-914); hence, a diagnosis of vitamin B12 deficiency and related pseudo-TMA was finally made. Since tests for anti-parietal cell antibodies and anti-intrinsic antibodies are not covered by the national health insurance system in Japan, these tests were not performed in the present case.

On the eighth hospital day, as the patient’s anemia symptoms had improved despite prolonged thrombocytopenia and newly demonstrated leukopenia (hemoglobin concentration: 8.8 g/dL; platelets: 48×10^9^/L; white blood cells: 3.2×10^9^/L; LDH: 1353 U/L; total bilirubin: 1.1 mg/dL), we discontinued the cyanocobalamin injections, and he was discharged from hospital. After discharge, we encouraged the patient to eat meat, which was effective at ameliorating his anemia. By five weeks after the initial diagnosis, his pancytopenia had improved, and his elevated LDH level had normalized (hemoglobin concentration: 13.1 g/dL; MCV: 91.5 fL; platelets: 219×10^9^/L; white blood cells: 10.2×10^9^/L; LDH: 211 U/L). By simply improving his diet, his vitamin B12 level normalized (375 pg/mL); hence, we concluded that these findings support a diagnosis of vitamin B12 deficiency due to dietary insufficiency, rather than antibody-related pernicious anemia. Furthermore, the schistocytes disappeared from his peripheral blood (Figure 1D), as did the abnormal distributions of the red blood cell and platelet histograms (Figures 3C, 3D).

## Discussion

TTP should initially be suspected in patients with thrombocytopenia and hemolytic anemia accompanied by schistocytosis. However, the differential diagnoses for TTP include hemolytic uremic syndrome, disseminated intravascular coagulation, malignant hypertension, collagenous diseases, pregnancy, and pseudo-TMA. It has been reported that 2.5% of cases of vitamin B12 deficiency exhibited schistocytosis and were diagnosed as pseudo-TMA [1]. As for the etiology of the vitamin B12 deficiency in these cases, 68.3% of the cases of vitamin B12 deficiency in pseudo-TMA were caused by pernicious anemia, 14.6% were caused by poor diet, 4.9% occurred in infants who were exclusively breastfed by mothers with pernicious anemia, 2.4% were caused by Helicobacter pylori-associated gastritis, and 2.4% were caused by ileocecal resection [7]. Our patient had no history of gastrointestinal surgery and was not taking any medications; hence, his preference to eat less meat was thought to be the main cause of his vitamin B12 deficiency. 

As for the pathophysiological mechanism of pseudo-TMA, it has recently been suggested that hyperhomocysteinemia, which is associated with endothelial damage, is one of its etiologies [8]. Hyperhomocysteinemia is caused by a combination of nutritional deficiency, particularly a deficiency of vitamin B6, vitamin B12, or folate, and a polymorphism of the methylenetetrahydrofolate reductase gene [8]. Clinically, hyperhomocysteinemia is a risk factor for thromboembolism because high plasma levels of homocysteine damage the vascular endothelium [8]. To distinguish between TTP and pseudo-TMA caused by vitamin B12 deficiency, it is necessary to measure ADAMTS13 activity and vitamin B12 levels. However, since it takes several business days for ADAMTS13 activity test results to become available, it is important to determine the distinguishing features of the condition based on general examination findings, the results of which can be obtained immediately.

It has been reported that compared with TTP, pseudo-TMA is associated with a decreased white blood cell count, thrombocytopenia, a high MCV, a low reticulocyte count, and a high LDH level [3]. To investigate schistocytosis, it has been recommended that the enumeration of fragmented red blood cells using an automated blood cell counter should be considered a useful complement to microscopic evaluation [9]. Recently, there was a report of a case in which a red blood cell histogram was useful for evaluating schistocytosis in a patient in whom mechanical red blood cell destruction had occurred due to venoarterial extracorporeal membrane oxygenation [10]. Our patient’s abnormal red blood cell histogram supplemented the detection of schistocytes in the peripheral blood under microscopy. In addition, the presence of megaloblasts in the peripheral blood made it possible to suspect vitamin B12 deficiency before the results of the bone marrow examination were available.

Previous studies have included cases in which unnecessary plasma exchange was performed because pseudo-TMA was misdiagnosed as true TTP [3]. Furthermore, two of these cases involved major complications, an anaphylactic transfusion reaction in one and hemothorax and cardiac arrest due to line placement in the other. In addition, there was a case in which plasma exchange was not effective, and a subsequent bone marrow examination revealed megaloblastic changes, leading to a diagnosis of vitamin B12 deficiency [11]. As for other diagnostic clues, hypersegmented neutrophils with more than six segments were seen in the present case, which have sensitivity and specificity values of 90% and 97%, respectively, for diagnosing vitamin B12 deficiency [12]. Recently, it has been reported that the PLASMIC score is useful for differential diagnosis and avoiding unnecessary plasma exchange in such cases [13]. However, as the patient’s PLASMIC score (five points) indicated that he was at intermediate risk, it is difficult to evaluate patients using this scoring system alone. In such circumstances, we suggest that a thorough review of the peripheral blood smear to confirm the presence of megaloblasts (namely, “megaloblastemia”) makes it possible to diagnose vitamin B12 deficiency quickly and inexpensively.

## Conclusions

We reported a case of pseudo-TMA caused by vitamin B12 deficiency in a 37-year-old male patient. When TTP is suspected because of hemolytic anemia accompanied by significant schistocytosis, it is important to consider pseudo-TMA due to vitamin B12 deficiency as a differential diagnosis. A thorough review of the peripheral blood film to detect megaloblasts and nuclear atypia in nucleated red blood cells is important for making a rapid and accurate diagnosis in such cases. In addition, red blood cell histograms can complement microscopic findings as a method for capturing changes in schistocytes over time.
